# Transduction of Lentiviral Vectors and *ADORA3* in HEK293T Cells Modulated in Gene Expression and Alternative Splicing

**DOI:** 10.3390/ijms26094431

**Published:** 2025-05-07

**Authors:** Yongqi Qian, Zhaoyu Liu, Qingqing Liu, Xiaojuan Tian, Jing Mo, Liang Leng, Can Wang, Guoqing Xu, Sanyin Zhang, Jiang Xie

**Affiliations:** 1 School of Basic Medical Sciences, Chengdu University of Traditional Chinese Medicine, Chengdu 611137, China; qq_yq12@163.com (Y.Q.); qingqingliue@163.com (Q.L.); tianxiaojuan_702@163.com (X.T.); 2 Institute of Herbgenomics, Chengdu University of Traditional Chinese Medicine, Chengdu 611137, China; liuzhaoyu2008@hotmail.com (Z.L.); jingmoe@163.com (J.M.); lling@cdutcm.edu.cn (L.L.); wangcan@cdutcm.edu.cn (C.W.); xuguoqing_0201@163.com (G.X.); 3 School of Chinese Materia Medica, Tianjin University of Traditional Chinese Medicine, Tianjin 300193, China; 4 Innovative Institute of Chinese Medicine and Pharmacy, Chengdu University of Traditional Chinese Medicine, Chengdu 611137, China

**Keywords:** HEK293T, RNA-seq, differentially expressed genes (DEGs), genes with differential transcript usage (gDTUs), *ADORA3*

## Abstract

For steady transgenic expression, lentiviral vector-mediated gene delivery is a commonly used technique. One question that needs to be explored is how external lentiviral vectors and overexpressed genes perturb cellular homeostasis, potentially altering transcriptional networks. In this study, two Human Embryonic Kidney 293T (HEK293T)-derived cell lines were established via lentiviral transduction, one overexpressing green fluorescent protein (GFP) and the other co-overexpressing GFP and *ADORA3* following puromycin selection to ensure stable genomic integration. Genes with differentially transcript utilization (gDTUs) and differentially expressed genes (DEGs) across cell lines were identified after short-read and long-read RNA-seq. Only 31 genes were discovered to have changed in expression when GFP was expressed, although hundreds of genes showed variations in transcript use. In contrast, even when co-overexpression of GFP and *ADORA3* alters the expression of more than 1000 genes, there are still less than 1000 gDTUs. Moreover, DEGs linked to *ADORA3* overexpression play a major role in RNA splicing, whereas gDTUs are highly linked to a number of malignancies and the molecular mechanisms that underlie them. For the analysis of gene expression data from stable cell lines derived from HEK293T, our findings provide important insights into changes in gene expression and alternative splicing.

## 1. Introduction

The process of modern drug development often begins with the exploration of ligands associated with established targets, like compounds that can bind to specific membrane protein receptors [1]. Cellular expression systems are essential in this procedure, facilitating rapid evaluation of how compounds interact with their targets and exploring the intricate mechanisms that govern these interactions, such as binding patterns and signaling pathways [2,3]. Additionally, with the fast-paced development of second-generation sequencing, the ability to conduct high-throughput RNA analysis enables researchers to capture the expression alterations of every gene concurrently [4]. Compared with the second-generation sequencing technology, the third-generation sequencing technology, with its long-read-length characteristic, can directly obtain the full-length transcript without complex splicing. It can more accurately identify alternative splicing isomers, detect new alternative splicing events, and avoid GC bias [5,6]. These approaches were quickly adopted for experiments with cell lines, resulting in substantial progress since its inception.

The use of lentiviral vectors for transduction is prevalent in cell line studies because it facilitates the creation of stable cell lines that express a specific external gene of interest [7,8]. As a vector, lentivirus is based on human immunodeficiency virus (HIV), which was initially used as a tool to study the biological characteristics of HIV, and then gradually developed into a gene transfer vector [9,10]. The second-generation lentivirus system consists of three plasmids: the transfer plasmid, the packaging plasmid, and the envelope plasmid [11,12]. Through lentivirus-mediated overexpression, the role of the target gene in cell proliferation, apoptosis, or some specific diseases can be clarified. The adenosine A3 receptor gene (*ADORA3*) was originally cloned in 1991 and subsequently identified as a subtype of the adenosine receptor [13,14]. *ADORA3* is widely expressed in a variety of tissues in the human body, including the brain, heart, lung, liver, kidney, and immune cells [15,16,17]. This widespread expression pattern is in accordance with the fact that *ADORA3* plays an important role in regulating a variety of physiological and pathological functions. *ADORA3* plays a role in immunomodulation, tumorigenesis and development, and neuroprotection, and it is important as a potential drug target for the development of new therapies to treat related diseases [18]. Studying gene function and signaling pathways is made easier by transfecting *ADORA3* in Human Embryonic Kidney 293T (HEK293T) cells. On the one hand, HEK293T cells have excellent transduction efficiency and protein expression levels of exogenous genes [19,20]. On the other hand, HEK293T cells have a relatively clear and stable genetic background, and their genomic features have been widely studied and deeply analyzed, which provides an ideal cell model for gene function studies [21,22]. On this basis, by overexpressing *ADORA3* in HEK293T cells, the interfering factors due to the complexity of the cellular genetic background can be effectively reduced so that the changes in the expression patterns of various genes triggered by the overexpression of *ADORA3*, as well as the specific mechanisms of its role in the regulation of intracellular signaling pathways and the cellular physiological activities, can be more precisely and clearly observed. This provides a theoretical basis and experimental foundation for exploring its potential mechanism of action in the development of tumors, inflammatory diseases, neurodegenerative diseases, and other diseases, and is of guiding significance for the development of therapeutic targets and novel therapeutic strategies for related diseases. In addition, the lentiviral vectors we used contained a green fluorescent reporter gene for assessing the efficiency of exogenous gene transduction. However, the random integration of green fluorescent protein (GFP) into the cellular genome may disrupt endogenous gene structures or regulatory elements, leading to insertion mutations that may cause inactivation of key genes or aberrant activation of genes. Therefore, the inclusion of GFP in this study is necessary.

To explore how plasmid transfection affects host cells, in our investigation, we developed two distinct cell lines derived from the HEK293T through the process of lentiviral vector transduction, where one line overexpressed the popular report gene encoding GFP (OE-GFP) and the other line overexpressed both GFP and *ADORA3* (OE-*ADORA3*). Subsequently, we conducted short-read and long-read RNA-seq on the cell lines to analyze the alterations in gene expression among them. In particular, our focus extended beyond merely assessing the variations in gene expression levels; we also examined the alterations in alternative splicing. The results indicated that overexpression of GFP and *ADORA3* had an impact on intracellular gene expression dynamics and alternative splicing processes. Overexpression of GFP affects a variety of genes related to the metabolism and synthesis of amino acids, whereas intracellular gene changes in HEK293T cells induced by overexpression of *ADORA3* are more enriched for a variety of malignant tumors and neurodegenerative disorders, which provides valuable insights for subsequent studies of GFP and *ADORA3*.

## 2. Results

### 2.1. Overexpression of GFP and ADORA3

**Figure 1:**
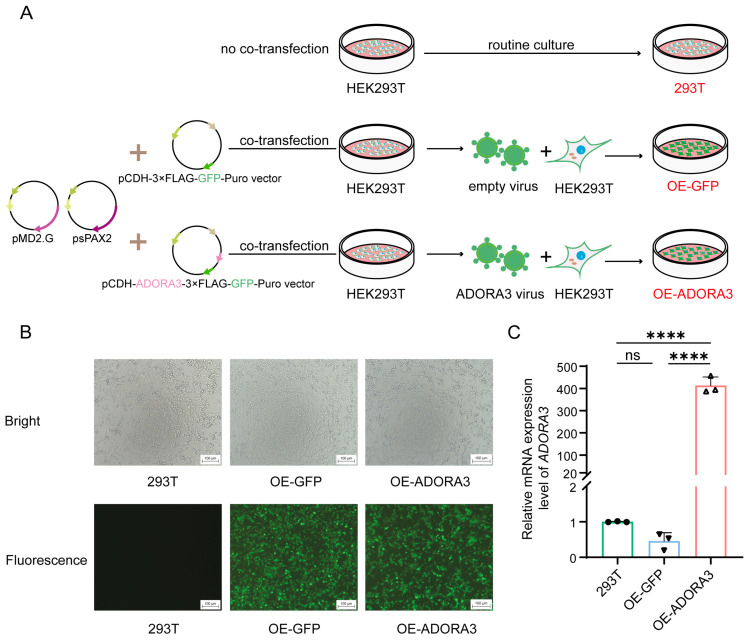
Overexpression of GFP and *ADORA3* in cell lines. (**A**) the illustration showing the process of routine culture of HEK293T cells and co-transfection of three plasmids to produce 293T, OE-GFP and OE-*ADORA3*; (**B**) observation of GFP fluorescence in cell lines; (**C**) testing of *ADORA3* mRNA level by RT-qPCR in 293T, OE-GFP and OE-ADORA3 (**** *p* < 0.0001, ns: not significant).

For clarity, the initial HEK293T cell line will be designated as 293T, the HEK293T cell line that has been altered to overexpress GFP will be called OE-GFP, and the HEK293T cell line that overexpresses both GFP and *ADORA3* will be referred to as OE-*ADORA3* (Figure 1A). Successful transduction was confirmed by examining GFP fluorescence using inverted fluorescence microscopy and measuring *ADORA3* mRNA levels in the cells through the RT-qPCR assay. The three groups of cells were observed under a microscope and inverted fluorescence microscopy, and it was found that OE-GFP and OE-*ADORA3* cells emitted green fluorescence under inverted fluorescence microscopy (Figure 1B). Afterwards, we detected *ADORA3* mRNA in the three groups of cells using RT-qPCR (Figure 1C). The outcomes showed that *ADORA3* mRNA was significantly elevated in OE-*ADORA3* (*p* < 0.0001). The aforementioned findings demonstrated the effective construction of OE-GFP and OE-*ADORA3*.

### 2.2. More than 2000 Genes Were Differentially Expressed in OE-ADORA3, While Only 31 Genes Were Differentially Expressed in OE-GFP

**Figure 2:**
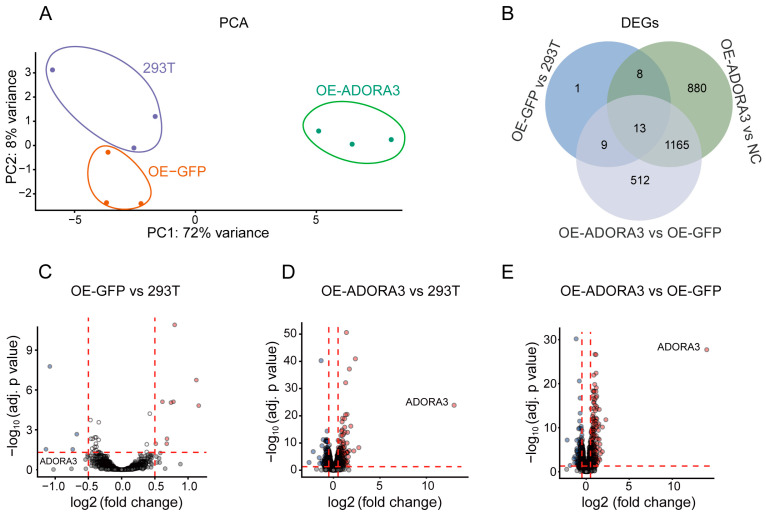
Translational alternations and date validation of DEGs between 293T, OE-GFP, and OE-*ADORA3*. (**A**) principal component analysis (PCA) based on TPM values of three replicates in each comparison; (**B**) Venn plot of genes from these three comparisons; volcano plots of the DEGs from the comparisons between (**C**) OE-GFP and 293T, (**D**) OE-*ADORA3* and 293T, (**E**) and OE-*ADORA3* and OE-GFP.

**Figure 3:**
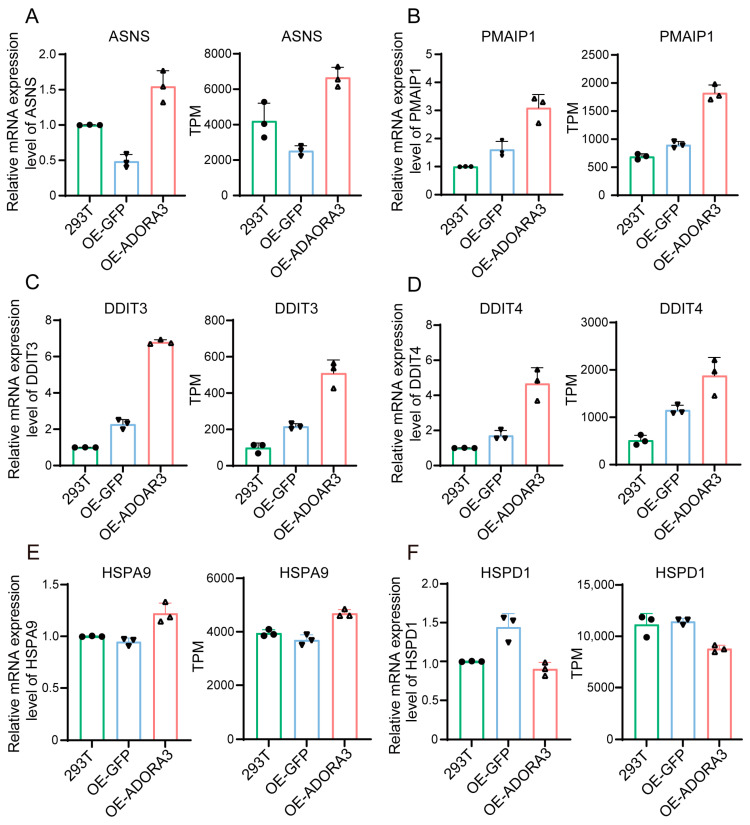
RT-qPCR validation and comparison with TPM of (**A**), ASNS (**B**), PMAIP1 (**C**), DDIT3 (**D**), DDIT4 (**E**), HSPA9 (**F**), and HSPD1.

We performed RNA-seq and transcriptome analysis on 293T, OE-GFP, and OE-*ADORA3*, each with three replicates. Utilizing principal component analysis (PCA) on the complete set of transcripts per million (TPM) values from all conditions and replicates made it evident that OE-*ADORA3* was distinguishable from 293T and OE-GFP in relation to the first principal component, while the second principal component allowed for differentiation between 293T and OE-GFP (Figure 2A). Subsequently, we conducted an analysis of differential expressions by comparing OE-GFP with 293T (Figure 2C), OE-*ADORA3* with 293T (Figure 2D), and OE-*ADORA3* with OE-GFP (Figure 2E). The comparison between OE-GFP and 293T revealed that 31 genes had altered expression levels, with 19 genes being upregulated and 12 genes being downregulated (Table S1). A comparison of OE-*ADORA3* with 293T revealed that 1084 genes had increased expression, while 982 genes had decreased expression. In a different comparison, OE-*ADORA3* versus OE-GFP showed that 876 genes were upregulated and 823 were downregulated (Tables S2 and S3). The Venn diagram indicated that over 50% of DEGs were common in the comparisons between OE-GFP and 293T, as well as OE-*ADORA3* and 293T, suggesting a consistent pattern in gene expression alterations (Figure 2B). Following the date’s RNA-seq transcriptomic analysis, three groups of common DEGs-ASNS, PMAIP1, DDIT3, DDIT4, HSPA9, and HSPD1 were randomly chosen for RT-qPCR verification in order to confirm the accuracy of the transcriptomic result. The results were consistent with the TPMs obtained by transcriptome analysis (Figure 3A–F).

**Figure 4:**
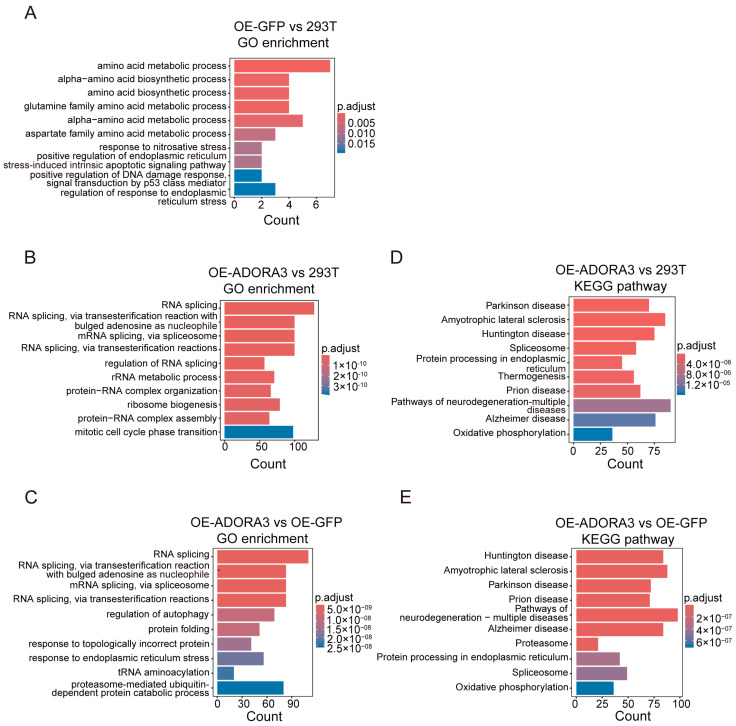
Functional enrichment of DEGs between 293T, OE-GFP, and OE-*ADORA3*. Top ten enriched GO terms of DEGs between (**A**) OE-GFP and 293T, (**B**) OE-*ADORA3* and 293T, and (**C**) OE-*ADORA3* and OE-GFP; top ten enriched KEGG pathways of DEGs between (**D**) OE-*ADORA3* and 293T and (**E**) OE-*ADORA3* and OE-GFP.

After that, functional enrichment analysis of DEGs was performed. The GO enrichment analysis revealed that amino acid metabolism, α-amino acid biosynthesis, and amino acid biosynthesis were the top ranked categories when comparing OE-GFP to 293T (Figure 4A and Figure S1A). RNA splicing and its subcategories were enriched when comparing OE-*ADORA3* to 293T, such as RNA splicing via transesterification reaction with bulged adenosine as a nucleophile, via transesterification reaction, and regulation of RNA splicing (Figure 4B and Figure S1B). Similarly, RNA splicing and its subcategories were also enriched when comparing OE-*ADORA3* with OE-GFP (Figure 4C and Figure S1C). These results showed a relation between the overexpression of *ADORA3* and the RNA splicing mechanism in the HEK293T cell line. In KEGG pathway enrichment analysis, the term spliceosome was among the top categories in the comparisons of OE-*ADORA3* with 293T and OE-*ADORA3* with OE-GFP, in accordance with the GO enrichment results. Several nervous system diseases were also among the top ranks in KEGG pathway enrichment analysis, such as Parkinson’s disease, Huntington’s disease, and Alzheimer’s disease (Figure 4D,E and Figure S2).

### 2.3. Detection of Differential Transcript Usage Among Cell Lines

Alternative splicing offered a different perspective for assessing the variations in translation among 293T, OE-GFP, and OE-*ADORA3* cell lines. Given that we have noted a significant enrichment of DEGs in RNA splicing, it is logical to delve deeper into the impact of alternative splicing in these cell lines.

**Figure 5:**
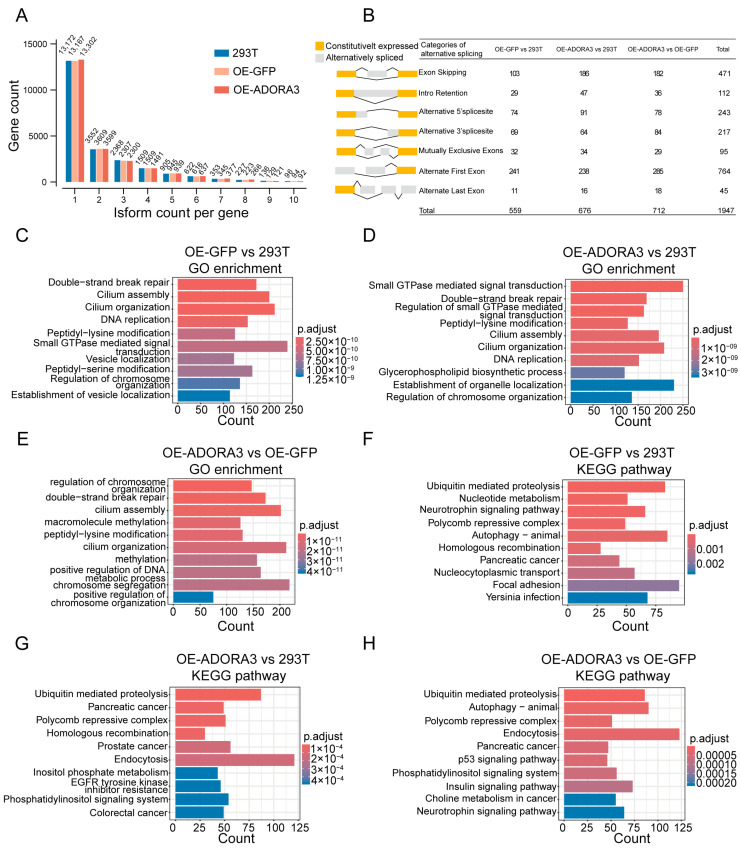
Alternative splicing events in 293T, GFP-293T, and A3AR-293T. (**A**) Number of genes with different isoform counts; (**B**) number of genes with different types of alternative splicing events; top ten enriched GO terms of gDTUs between (**C**) OE-GFP and 293T, (**D**) OE-*ADORA3* and 293T (**E**), and OE-*ADORA3* and OE-GFP; top ten enriched KEGG pathways of gDTUs between (**F**) OE-GFP and 293T (**G**), OE-*ADORA3* and 293T (**H**), and OE-*ADORA3* and OE-GFP.

**Figure 6:**
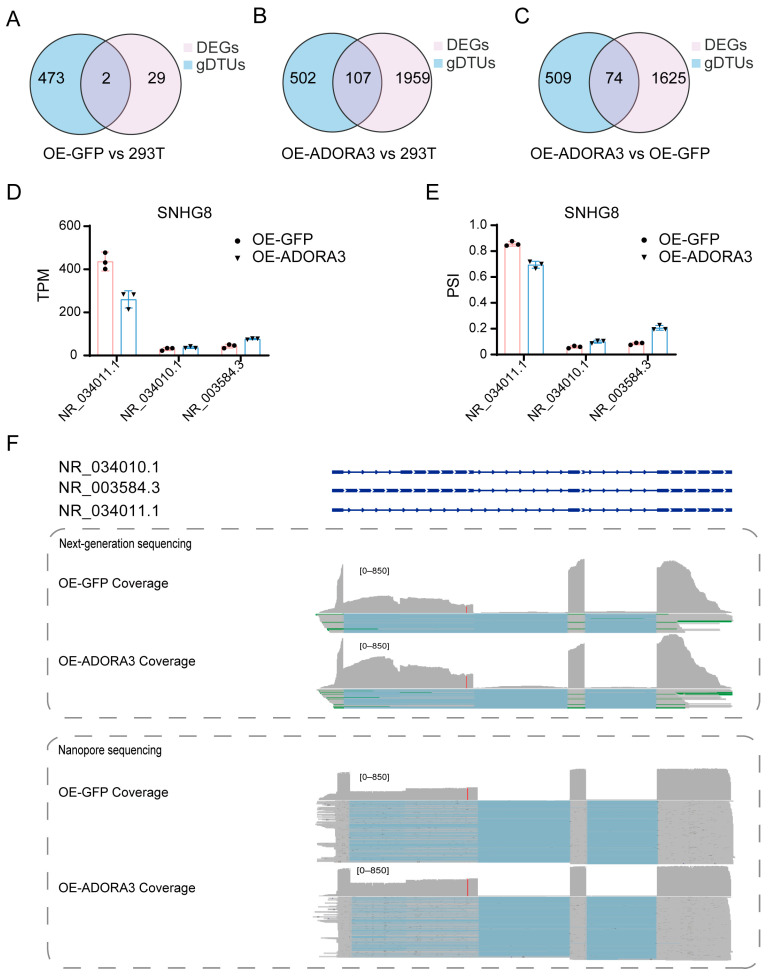
The intersection between DEGs and gDTUs. Venn plots of DEGs and gDTUs in comparisons between (**A**) OE-GFP and 293T, (**B**) OE-*ADORA3* and 293T, (**C**) and OE-*ADORA3* and OE-GFP; (**D**) TPM values of SNHG8 across three replicates in OE-GFP and OE-*ADORA3*; (**E**) TPM proportion of NR_034011, NR_034010, and NR_003584 in OE-GFP and OE-*ADORA3*; (**F**) the gene structure and RNA-seq read depth of NR_034011, NR_034010, and NR_003583 visualized in IGV in OE-GFP and OE-*ADORA3*.

We first examined isoform counts of genes in three cell lines. The overall patterns were quite similar in 293T, OE-GFP, and OE-*ADORA3*, with ~13,000 expressed genes having only one isoform, while ~10,000 genes had more than one (Figure 5A). Using the standard Suppa pipeline, we further identified genes with differential transcript usage (gDTUs) in the comparisons of cell lines. In our analysis of gDTUs, we identified 559, 676, and 712 events (475, 609, and 583 genes) as gDTUs in the comparisons between OE-GFP and 293T, OE-*ADORA3* and 293T, and OE-*ADORA3* and OE-GFP, respectively (Figure 5B and Figure 6A–C). This inconsistency pointed to the possibility that alternative splicing could fulfill a different role compared to the amount of gene expression. The selection of an alternative initial exon, which affects the selection of cis-regulatory elements like promoters, represents the primary event of alternative splicing, with exon skipping being the next most common one (Figure 5B).

We next performed functional enrichment analysis of gDTUs among the three comparisons. A comparison of OE-GFP and 293T showed that GO terms were significantly enriched in categories such as double-strand repair, cilium assembly, cilium organization, and DNA replication (Figure 5C and Figure S3A). When comparing OE-*ADORA3* with 293T, the most abundant categories comprised small GTPase-mediated signal transduction and its regulation, double-strand repair, peptidyl-lysine modification, and cilium assembly and organization (Figure 5D and Figure S3B). Lastly, in the OE-*ADORA3* and OE-GFP comparison, regulation of chromosome organization, double-strand repair, cilium assembly and regulation, macromolecule methylation, and peptidyl-lysine modification were the top-ranked GO terms (Figure 5E and Figure S3C). Global observations revealed that the first 10 GO terms were very similar in the OE-GFP and 293T group and the OE-*ADORA3* and 293T group, except for the different order. Furthermore, GO terms such as double-strand repair, cilium assembly, and cilium regulation were enriched in all three groups (Figure 5C–E). The above results suggest that when the internal environment of the HEK293T cell line is changed, the gene splicing associated with these GO terms may be altered and affect the most basic functions of the cell in the first place. It is this change in associated gene splicing that makes the KEGG pathways enriched under different changes not identical. The results of KEGG pathway enrichment had an obvious feature indicating that some tumor-related pathways were involved. In a three-group comparison, the pancreatic cancer pathway was one of the top 10 enriched KEGG pathways (Figure 5F–H and Figure S4). In the comparison between OE-*ADORA3* and 293T, three cancer pathways were among the top 10, including pancreatic cancer, prostate cancer, and colorectal cancer (Figure 5G and Figure S4B). In addition, several cancer-related pathways had occasionally occurred, e.g., homologous recombination, EGFP tyrosine kinase inhibitor resistance, and the p53 signaling pathway. DEGs of these comparisons were functionally enriched in both splicing and nervous system diseases, while gDTUs were functionally enriched in some cancer-related GO terms or pathways. These findings might imply that the HEK293T cell line functions as a system where splicing-related genes are susceptible to regulation, which in turn could alter the alternative splicing configurations of genes associated with cancer.

### 2.4. The SNHG8 Gene Represents an Example of Both DEGs and gDTUs

The discrepancy in number and functional enrichment of DEGs and gDTUs implied that DEGs and gDTUs might only have limited connections. When comparing OE-GFP with 293T, there were only two common genes in 31 gDTUs and 475 DEGs and, comparing OE-*ADORA3* with OE-GFP, 74 common genes in 583 gDTUs and 1699 DEGs (Figure 6A,C; Tables S1, S3, S4 and S6). This small number in OE-GFP and 293T may result from the fact that the number of DEGs is small, but even under the comparison of OE-*ADORA3* versus 293T, in which the number of DEGs is more than 2000, the number of overlapped DEGs and gDTUs was 107, accounting for about five percent of all DEGs and less than twenty percent of gDTUs (Figure 6B; Tables S2 and S5). Thus, DEGs and gDTUs could be two related groups, but they would largely play different roles.

We further conducted a detailed examination of SNHG8, which was identified as both a DEG and a gDTU when comparing OE-GFP with OE-*ADORA3*. SNHG8, small nucleolar RNA host gene 8, is a gene that encodes a long non-coding RNA, which could be further processed into small nucleolar RNAs [23]. This gene is highly expressed in many tissues, such as the ovary and cervix, according to GTEx data (2013). The overall expression level of SNHG8 dropped from 509.3 in OE-GFP to 373.1 in OE-*ADORA3*, indicating that this gene is a DEG between the two conditions (Figure 6D). SNHG8 has three isoforms, i.e., NR_034011, NR_034010, and NR_003583 (Figure 6E,F and Figure S5). Comparing with NR_034011, the shortest isoform, NR_034010 has an additional exon, while NR_003583’s first exon is much longer. In addition, NR_003583 is the intron-retention form of NR_034010. While NR_034011 was the most prevalent isoform regarding expression levels, its proportion of overall SNHG8 expression fell from 85.7% in OE-GFP to 69.8% in OE-*ADORA3*. In contrast, the expression calculated as TPM of NR_003583/NR_034010 increased from 43.5/30.0 to 76.5/36.8. Thus, the calculated delta-PSI (percent spliced in) values for NR_034011, NR_003583, and NR_034010 were -0.16, 0.12, and 0.04, respectively, with corresponding *p*-values of 0.0030, 0.0030, and 0.02. Oxford Nanopore technology (ONT) reads were also visualized using Integrative Genomics Viewer (IGV, 2.18.4), and it was found that three isoforms of SNHG8 were expressed in accordance with the results obtained from short reads (Figure 6F). SNHG8 serves as an example of a gene that qualifies as both a DEG and a gDTU, given that its multiple isoforms show varying patterns of increase or decrease when comparing two different states.

## 3. Discussion

Gene delivery using lentiviral vectors is a cornerstone of molecular biology research, especially for achieving stable integration and sustained expression of transgenes like *ADORA3* [24,25]. Factors affecting the efficiency of lentiviral transduction—including vector design, promoter strength, and host cell compatibility—play a key role in regulating transcriptional outcomes and post-transcriptional regulation [26,27,28]. In this study, we used the second-generation lentiviral system pCDH to overexpress GFP and *ADORA3* in HEK293T cells. The results revealed that overexpression of GFP and *ADORA3* affected gene expression and variable shear in HEK293T cells, and there was a dual regulation of transcriptional and posttranscriptional activities. It should be noted that there is a methodological limitation in this study. Specifically, biological replicates were not set up in the virus transduction experiment. Although we verified the reproducibility of the transcriptome data through three independent RNA library construction sequencing, the potential insertion mutation effect of the integrated lentiviral vector may still affect the gene expression patterns of some cells [29].

mRNA is a key intermediate product of gene expression, which transcribes and transmits the genetic information of DNA to the ribosome for translation to generate proteins [30]. mRNA stability, translation efficiency, and splicing patterns directly affect the level of gene expression and protein diversity [31,32,33]. The use of RNA-seq enables high-throughput, high-precision quantitative analysis of genome-wide gene expression [34]. Right now, second-generation sequencing platforms are the most favored, offering both high throughput and a low rate of mistakes. They can provide high-confidence sequence information that can be used for the genome, transcriptome, epigenome, and other areas of study [35,36]. However, their short read length leads to difficulties in assembling complex genomes and in accurately detecting structural variants and alternative splicing events in long fragments, and GC bias may introduce uneven coverage problems [37]. With its ultra-long read length, ability to detect RNA sequences and their methylation modifications directly, and lack of GC bias, third-generation sequencing technology is a powerful tool, compared with second-generation sequencing technology. It can be used for complex genome assembly, structural variation analysis, full-length transcript isomer analysis, and the detection of epigenetic modifications [38,39]. By comparing transcriptome data before and after the overexpression of genes, differentially expressed genes can be identified, providing insight into the pattern of changes in gene expression [40,41]. In this study, GFP and *ADORA3* were activated by host cells through the transcription process of lentiviral vectors, resulting in the specific expression of these exogenous genes. As a commonly used reporter gene, GFP is frequently thought of as an “inert” labeling tool. Recent research, however, has revealed that GFP overexpression may disrupt host cell transcriptomes in a number of ways. It has been shown that GFP is made up of 238 amino acids, and the enormous volume of synthesis depletes the free amino acid pool, resulting in limited synthesis in the host cell [42]. Overexpression of GFP results in alternations in aspartate, glutamate, and glutamine, which is consistent with our analysis of changes in DEGs in overexpressed GFP [43,44]. Furthermore, reactive oxygen species are produced when GFP is excited by blue light during fluorescence imaging, damaging DNA and impairing replication integrity [45]. In summary, although the OE-GFP group uses the same lentiviral vector system and transduction process as the OE-*ADORA3* group, which allows the OE-GFP group to reflect, to some extent, the general effects of the lentiviral vector transduction process on the cells, as well as to serve as a control group to assess changes in the OE-*ADORA3* group, the presence of GFP itself on cells suggests that the OE-GFP group may not be adequate as a control group to assess the specific effects induced by the OE-*ADORA3* group.

When *ADORA3* was overexpressed, thousands of genes were up- or downregulated; these genes were functionally enriched in a variety of functions related to RNA splicing and involved in a variety of neurodegenerative diseases, as well as oxidative phosphorylation. The pathogenesis of neurodegenerative diseases is complex, usually involving the interaction of multiple factors and multiple pathways, which ultimately leads to the progressive loss of neuronal structure and function. Mitochondrial dysfunction is a common degenerative feature of some neurodegenerative diseases [46,47,48,49,50]. The KEGG pathway corresponding to DEGs between OE-*ADORA3* and 293T and between OE-*ADORA3* and OE-GFP include oxidative phosphorylation, which is one of the core processes of mitochondrial function. The dysfunction of oxidative phosphorylation will significantly affect the function of mitochondria, thus affecting the energy metabolism and survival of cells. In Parkinson’s disease, the activity of mitochondrial complex I is reduced, affecting the function of the electron transport chain and insufficient ATP production, ultimately leading to neuronal dysfunction and death. This suggests that *ADORA3* may play a role in Parkinson’s disease and has potential as a therapeutic target. Mitochondrial complex III is associated with Alzheimer’s disease, and its abnormality may lead to excessive production of reactive oxygen species, which can trigger oxidative stress and damage cell structure and function. The common DEGs, HSPA9 and HSPD1, in the three groups verified in the previous section are all involved in maintaining protein homeostasis in mitochondria, preventing protein misfolding and aggregation. Their expression levels correlate with the progression of Parkinson’s disease and neurodegenerative diseases [51,52]. In addition, in the comparison between OE-*ADORA3* and OE-GFP, enrichment of the DEG-associated KEGG pathways was found to be associated with insulin resistance. Insulin resistance is thought to be the culprit of Alzheimer’s disease characteristics caused by neuroinflammation and oxidative stress, and Parkinson’s disease and Huntington’s disease are also strongly associated with insulin resistance [53]. These findings further suggest *ADORA3*’s relevance to neurodegenerative diseases and its potential as a therapeutic target. Future studies for neurodegenerative diseases could be conducted in the direction of the molecular mechanism of *ADORA3* and oxidative phosphorylation, for example, whether it affects the oxidative phosphorylation process by regulating the mitochondrial membrane potential, the activity of the electron transport chain complex, or the function of ATP synthase. Validation in appropriate neural cell and animal models and further studies can be performed to investigate the relationship between *ADORA3*-mediated improvement of oxidative.

In contrast to DEG, in the analysis of gDTU, we found that the alternative splicing of hundreds of genes was influenced between any two conditions. In addition, when comparing OE-*ADORA3* with both 293T and OE-GFP, multiple cancer-related pathways were enriched in KEGG pathway analysis. Previous studies have found that *ADORA3* is highly expressed in a variety of malignant tumors, including melanoma, breast cancer, prostate cancer, liver cancer, pancreatic cancer, lung cancer, lymphoma, glioblastoma, and malignant pleural mesothelioma [54,55,56]. The high expression of *ADORA3* in malignant tumors promotes the occurrence, development, metastasis, and other processes of tumors, such as accelerating the deterioration of tumors by promoting the proliferation of tumor cells, facilitating tumor immune escape, and promoting tumor angiogenesis. However, in recent years, research has revealed the opposite phenomenon, making *ADORA3*’s role in cancer complicated and contradictory. *ADORA3* has a dual effect on the proliferation of malignant tumor cells. In one study, the activation of *ADORA3* with the *ADORA3* agonist N6-(3-iodobenzyl) adenosine-5′-n-methylureamine inhibited adenylate cyclase in AT6.1 rat prostate cancer cells and thus inhibited PKA-mediated ERK1/2 activation, leading to a reduction in prostate cancer cell proliferation [57]. However, PKA inhibition resulting from decreased adenylate cyclase activity has been found in melanoma and liver cancer cells to increase glycogen synthesis kinase 3β, thus promoting cell proliferation [57]. In this study, the signaling pathway mentioned above was not enriched in KEGG pathway analysis, but the phosphatidylinositol signaling system was enriched. The phosphatidylinositol signaling system can activate ERK1/2 through PKC and down-regulate the activity of glycogen-synthesizing kinase 3β through the PI3K/Akt pathway. However, its effect on cell proliferation needs further research. From the above, we speculated that the promoting/inhibiting effect of *ADORA3* is likely to be affected by agonist concentration, agonist type, and cell type. The use of 2-chloro-N6-(3-iodophenyl)-adenosine-5′-n-methyluridine, an agonist with high affinity for *ADORA3*, induces apoptosis of Hep-3B cells in vitro and in vivo by regulating the PI3K-NF-κB signaling pathway [57]. In the analysis of DEGs, we focused on the genes *HSPA9* and *HSPD1*, which influence tumor cell survival by regulating the stability of the mitochondrial membrane and the conformation and thus function of mitochondrial proteins [58]. The enrichment of apoptosis–multiple species in KEGG pathway analysis was also observed in the comparison of OE-*ADORA3* with 293T and OE-GFP. In the study of the role of *ADORA3* in cancer development, as well as therapy, concentration of agonist action should be confirmed to avoid the situation of tumor-promoting proliferation because of the dual action of *ADORA3*. *ADORA3* mediates multiple signaling pathways, and the specific mechanism of action of each signaling pathway affecting the proliferation, apoptosis, invasion, and metastasis of cancer cells should be clarified. In addition, it is necessary for the changes in alternative splicing induced by *ADORA3* to be included in studies. In this study, we found that not only gene expression quantity alternation but also alternative splicing may contribute to the role *ADORA3* may play in tumors. Our findings uncovered an unexpected insight that *ADORA3* could influence tumors not just by altering the expression levels of certain genes but also by affecting their alternative splicing patterns. Therefore, it is worth incorporating alternative splicing analysis into future research related to *ADORA3*, examining both cell lines and in vivo experiments.

## 4. Materials and Methods

### 4.1. Generation of Genetically Modified HKE293T Cells

HEK293T cells were purchased from Procell Life Science & Technology Company (Wuhan, China). HEK293T were grown in Dulbecco’s modified eagle medium (DMEM) (Gibco^®^, Waltham, MA, USA) supplemented with 10% fetal bovine serum (VivaCell, Shanghai, China) and 1% penicillin-streptomycin (Gibco^®^, Waltham, MA, USA) at 37 °C and 5% CO_2_. The transfer vector, pCDH-3×Flag-GFP-Puro or pCDH-*ADORA3*-3 × Flag-GFP-Puro (Youbio, Changsha, China), along with the psPAX2 (Youbio, Changsha, China) packaging vector and the pMD2.G (Youbio, Changsha, China) envelope vector, were co-transfected into HEK293T cells to produce the lentivirus with Lipofectamine 3000 (Invitrogen, Carlsbad, CA, USA) according to the manufacturer’s protocol [59]. The supernatants were harvested and filtered using a 0.45 µm bottle-top filter at 48 and 72 h post-transfection to obtain the packaged lentivirus. The packaged lentivirus was then transduced into cultured HEK293T cells [60]. After a 48 h incubation post-transduction, the infection efficiency was assessed by observing GFP fluorescence under inverted fluorescence microscopy [60]. The expression of *ADORA3* was confirmed using the quantitative reverse transcription polymerase chain reaction (RT-qPCR) method. Finally, the cells exhibiting high infection efficiency and overexpressed *ADORA3* were selected for downstream analysis.

### 4.2. RNA Extraction and RT-qPCR

Total RNA was extracted from diverse cells, including HEK293T cells and those harboring either an empty vector or overexpressed *ADORA3* plasmid, using the RNAiso Plus (Takara, Tokyo, Japan) [61,62]. The integrity and concentration of the total RNA were evaluated through 1% agarose gel electrophoresis and NanoDrop™ One Spectrophotometry (Invitrogen, Wilmington, DE, USA). A total of 1 μg total RNA was employed for reverse transcription analysis, using the Primer Script Fast RT Reagent Kit with gDNA eraser (RR092S) (Takara, Tokyo, Japan).

RT-qPCR was subsequently employed to assess the expression level of *ADORA3* and other differentially expressed genes (DEGs) in the cells. Briefly, RT-qPCR assays were executed on the QuantStudio™ 5 Real-Time PCR detection system (Thermo Fisher Scientific, Waltham, MA, USA) using TB green premix EX Tap II fast qPCR (Takara, Tokyo, Japan). The primers for these RT-qPCR assays were synthesized by Sangon Biotech (Shanghai, China), with their sequences detailed in Table 1. Glyceraldehyde-3-phosphate dehydrogenase (*GAPDH*) was used as the internal reference gene. The 2^−ΔΔCT^ method was applied to normalize the expression of each transcript to the *GAPDH* mRNA level [63,64]. The experiments were repeated at least three times. Statistical analysis was performed using one-way ANOVA in GraphPad Prism 10.0 software (San Diego, CA, USA) to determine the significance of differences in gene expression.

### 4.3. RNA Library Construction and Sequencing

HEK293T cells, as well as those expressing either an empty vector or an overexpressed *ADORA3* plasmid, were harvested for RNA sequencing. Total RNA was extracted using RNAiso Plus (Takara, Tokyo, Japan). The amplification products were cycled to generate single-stranded DNA libraries using the MGIEasy Circularization Kit (Cat# 1000004155) and then sequenced on the DNBSEQ-T7 platform (BGI, Shenzhen, China). Moreover, the cDNA–polymerase chain reaction (PCR) Sequencing Kit (SQK-PCS109) was used to perform cDNA sequencing on a PromethION sequencer (ONT, Oxford, UK). After sequencing, low-quality, raw sequencing reads were filtered to obtain clean data and used for subsequent analysis.

### 4.4. RNA-Seq Data Analysis

Salmon (version 0.14.2) was used for quantification at the transcript level with BGI clean reads in fastq format (−1 ISF —gcBias), and the quantification results were converted to the gene level using txtimport (1.30.0); then subsequent analysis was performed with DESeq2 (1.42.0) [40,65,66]. Gene enrichment analysis was performed using clusterProfiler (4.10.0), and all DEGs with *p*-value < 0.05 were used for enrichment analysis [67]. Gene Ontology (GO) enrichment was conducted using the default parameters of clusterProfiler, and KEGG pathway enrichment was performed with a *p*-value cutoff = 0.05. Volcano plots were generated using *p*-value < 0.05 and log2 fold change > 0.5.

### 4.5. Alternative Splicing Analysis

AS analysis was performed on short reads using Suppa2 (2.3), with generateEvents generating ioi and ioe files, psiPerIsoform and psiPerEvent calculating the psi for isoforms and events, respectively, and diffSpice calculating delta-PSI [68]. We used the total number and types of events for a single gene to represent the total number and types of AS for the entire gene. We filtered the final results to retain only the AS events where at least two genes occurred. For the enrichment of genes with AS events, delta-PSI was used instead of log2 fold change for the enrichment analysis. GO enrichment was performed using the default parameters of clusterProfiler, and Kyoto Encyclopedia of Genes and Genomes (KEGG) pathway enrichment was not filtered by *p*-value. The ONT full-length reads were corrected by fmlrc2 with BGI reads and then mapped to reference transcriptome sequences using Minimap2(–ax splice —MD —cs = long, version 2.28) [69,70]. Only BGI reads used for quantification and the ONT reads were used to verify the AS. The structural information and the sequencing data of the focal gene generated by short reads and long reads were visualized on the IGV 2.18.4 software [71].

### 4.6. Statistical Analysis

Differences between HEK293T cells, overexpression of GFP, and *ADORA3* were evaluated using one-way analysis of variance (ANOVA). The results of each experiment were recorded as three replicates of the mean ± SD. The data were analyzed by GraphPad Prism 10.0 software. *p* < 0.05 was considered statistically significant.

## 5. Conclusions

In summary, based on the above observations, we concluded that both the infection of lentivirus and overexpression of external genes may affect the transcription landscape of the HEK293T cell line. The initial stage of drug discovery often involves experiments with cell lines, and analyzing the transcriptional activity of these lines in response to compounds may reveal important information for target identification [3]. As a result, attention must be given to the effects of external factors on the transcriptional dynamics within cell lines [72]. Moreover, the alternation of gene expression quantity and the gDTUs may have different impacts on the transcriptional dynamics of specific cell lines, as revealed in this study. Overexpression of *ADORA3* primarily leads to the identification of DEGs that are significantly linked to neurodegenerative conditions, while gDTUs are more frequently associated with multiple pathways related to cancer. This may be because alternative splicing is an important regulatory factor in the development of cancer [73,74,75]. Future research on *ADORA3* may thus concentrate more on the connection between *ADORA3* and the aforementioned illnesses. Based on this work, tailored medication development can precisely identify drug-induced particular modifications impacting target genes. Furthermore, it is important to consider the impact of GFP on host cells. To rule out GFP-induced alterations, comparable control groups should be established for research pertaining to reactive oxygen species and DNA replication. Our findings support the use of HEK293T cells for drug screening and aid in the analysis of the particular changes brought on by overexpressed genes. Drug development based on cell-based research will require a thorough examination of several facets of gene expression, such as variations in expression levels or alternative splicing [76,77]. Future research should incorporate biological replicates and select an appropriate control group to assess the specific impact of viral transduction and specific genes on gene expression profiles more comprehensively and accurately, which will help to mitigate the potential biases caused by the current lack of biological replicates and lead to more reliable and robust results.

## Figures and Tables

**Table 1:** The primers for RT-qPCR analysis of DEGs.

Primer	Sequence (5′-3′)
GAPDH-F	GGAGCGAGATCCCTCCAAAAT
GAPDH-R	GGCTGTTGTCATACTTCTCATGG
ADORA3-F	GCTGGTCATCTGCGTGGTCAAG
ADORA3-R	GGATTGTGATGCCCAGGCTGAC
PMAIP1-F	ACTCACCGTGTGTAGTTGGC
PMAIP1-R	CACTCGACTTCCAGCTCTGCT
ASNS-F	GGAAGACAGCCCCGATTTACT
ASNS-R	AGCACGAACTGTTGTAATGTCA
DDIT4-F	TGAGGATGAACACTTGTGTGC
DDIT4-R	CCAACTGGCTAGGCATCAGC
DDIT3-F	GGAACCTGAGGAGAGAGTGTTC
DDIT3-R	CTGCCATCTCTGCAGTTGGA
HSPA9-F	ACCTGCTGATGAGTGCAACA
HSPA9-R	AGTGCCAGAACTTCCAGAGC
HSPD1-F	ACGACCTGTCTCGCCG
HSPD1-R	AATCGTAGCAACCTGTGCAA

## Data Availability

The data presented in this study are openly available from the National Genomics Data Center at https://ngdc.cncb.ac.cn/bioproject/browse/PRJCA036438 (accessed on 21 February 2025), reference number PRJCA036438.

## Supplementary Materials

The following supporting information can be downloaded at https://www.mdpi.com/article/10.3390/ijms26094431/s1.

## Abbreviations

The following abbreviations are used in this manuscript:

HEK293T	Human Embryonic Kidney 293T
gDTUs	Genes with differential transcript usage
GFP	Green fluorescent protein
ADORA3	Adenosine A3 receptor
HIV	Human immunodeficiency virus
DMEM	Dulbecco’s modified eagle medium
RT-qPCR	Quantitative reverse transcription polymerase chain reaction
DEGs	Differentially expressed genes
GAPDH	Glyceraldehyde-3-phosphate dehydrogenase
PCR	Polymerase chain reaction
GO	Gene Ontology
KEGG	Kyoto Encyclopedia of Genes and Genomes
PCA	Principal component analysis
TPM	Transcripts per million
PSI	Percent spliced in
ONT	Oxford Nanopore technology
IGV	Integrative Genomics Viewer
