# Supplementary material for: Transduction of Lentiviral Vectors and ADORA3 in HEK293T Cells Modulated in Gene Expression and Alternative Splicing
Source: Int J Mol Sci. 2025 May 7;26(9):4431. doi: 10.3390/ijms26094431 (PMC12072217; doi:10.3390/ijms26094431)
Supplement: Supplementary file 1 [file ijms-26-04431-s001.zip › Supplement Materials.pdf]

Supplementary Materials for AS

Supplement Figures

Figure. S 1 Top twenty enriched GO terms of DEGs. 2
Figure. S 2 Top twenty enriched KEGG pathways of DEGs. 3
Figure. S 3 Top fifty enrichment GO terms of gDTUs 4
Figure. S 4 Top fifty enriched KEGG pathways of gDTUs. 5
Figure. S 5 SNHG8's sashimi plot in OE-GFP and OE-ADORA3. 6

Supplement Tables

Table. S 1 DEGs in OE-GFP vs 293T 7
Table. S 2 DEGs in OE-ADORA3 vs 293T 7
Table. S 3 DEGs in OE-ADORA3 vs OE-GFP 7
Table. S 4 gDTU in OE-GFP vs 293T 7
Table. S 5 gDTU in OE-ADORA3 vs 293T 7
Table. S 6 gDTU in OE-ADORA3 vs OE-GFP 7

Supplement Figures

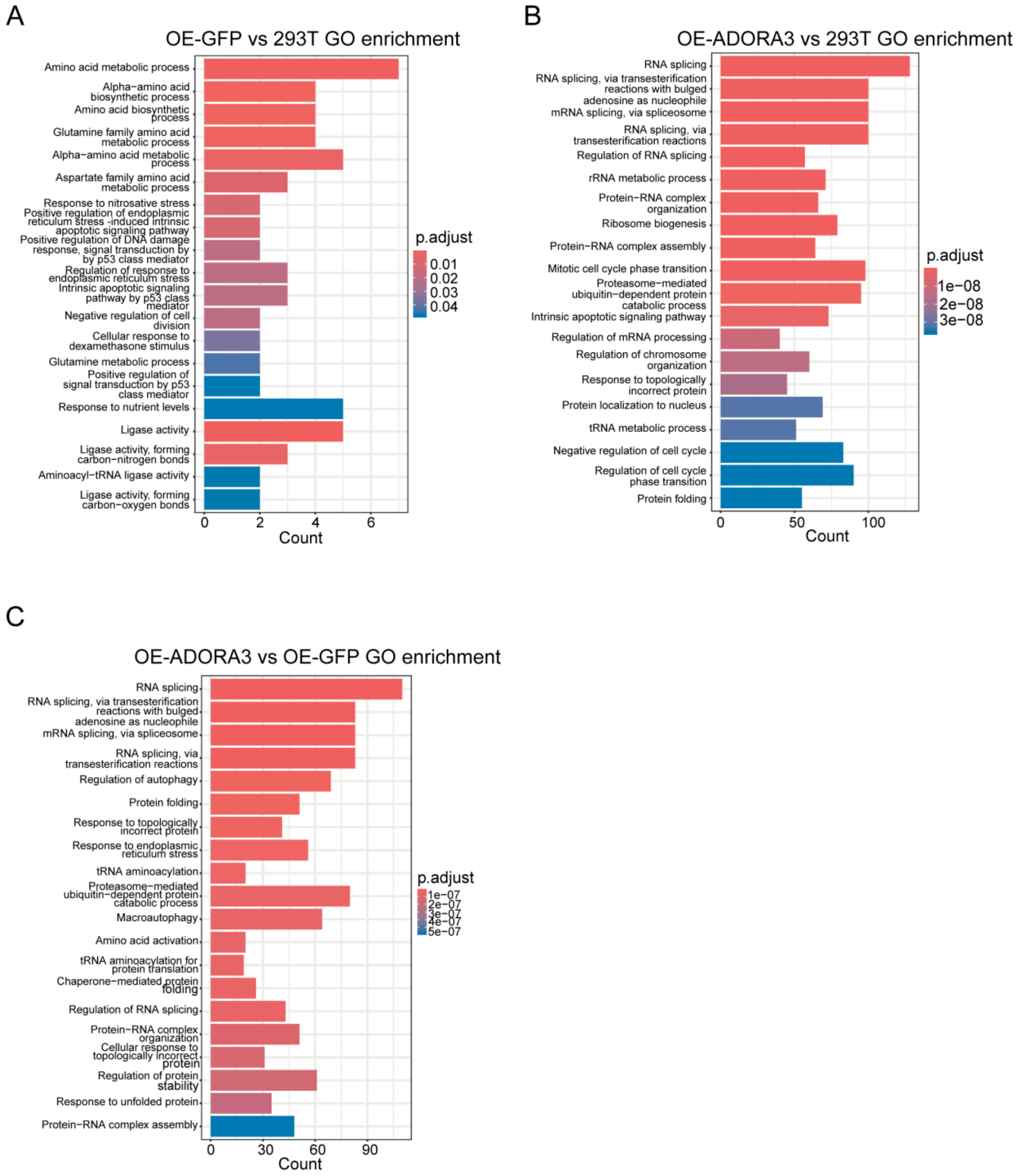

Figure. S 1 Top twenty enriched GO terms of DEGs between (A), OE-GFP and 293T (B), OE-ADORA3 and 293T (C), OE-ADORA3 and OE-GFP.

A

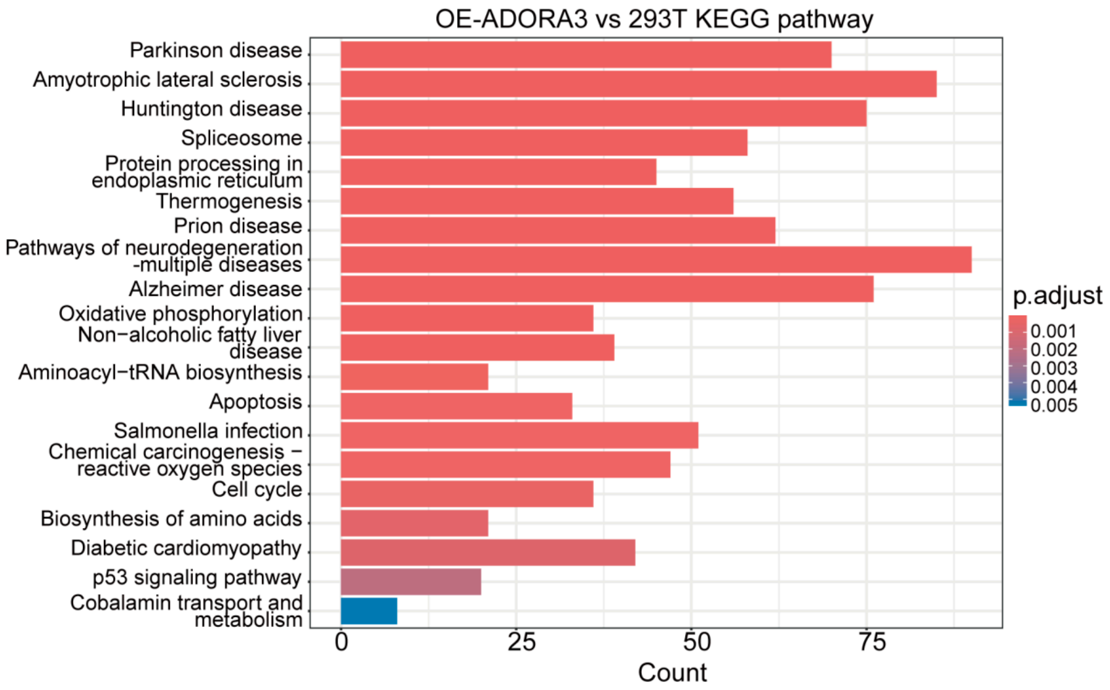

B

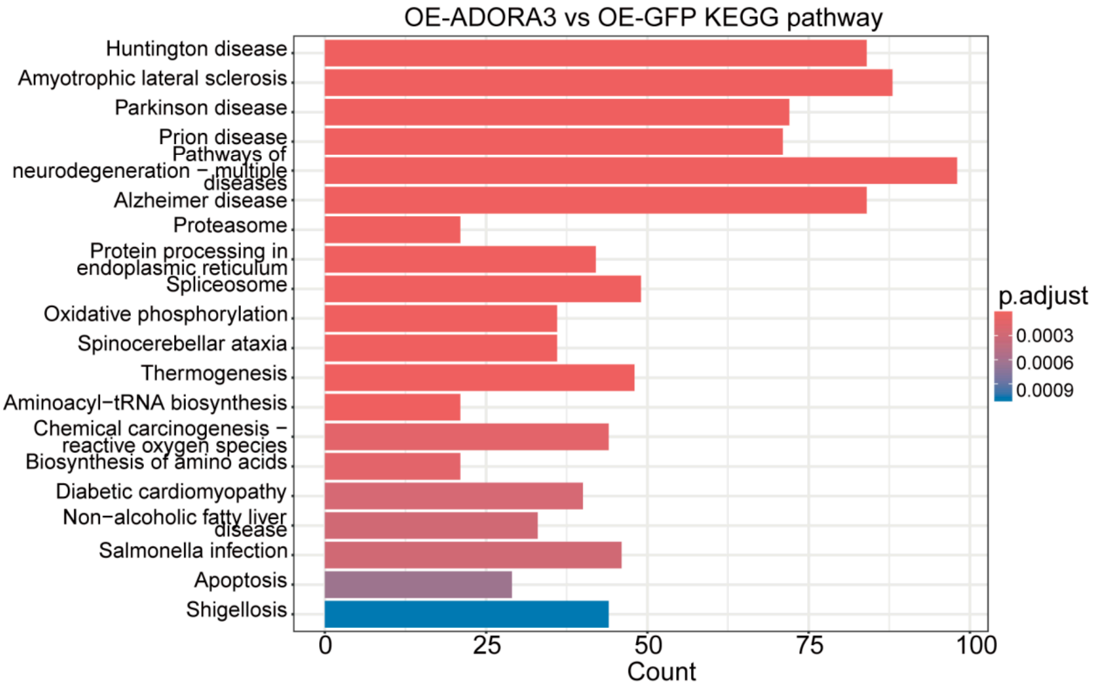

Figure. S 2 Top twenty enriched KEGG pathways of DEGs between (A), OE-ADORA3 and 293T (B), OE-ADORA3 and OE-GFP.

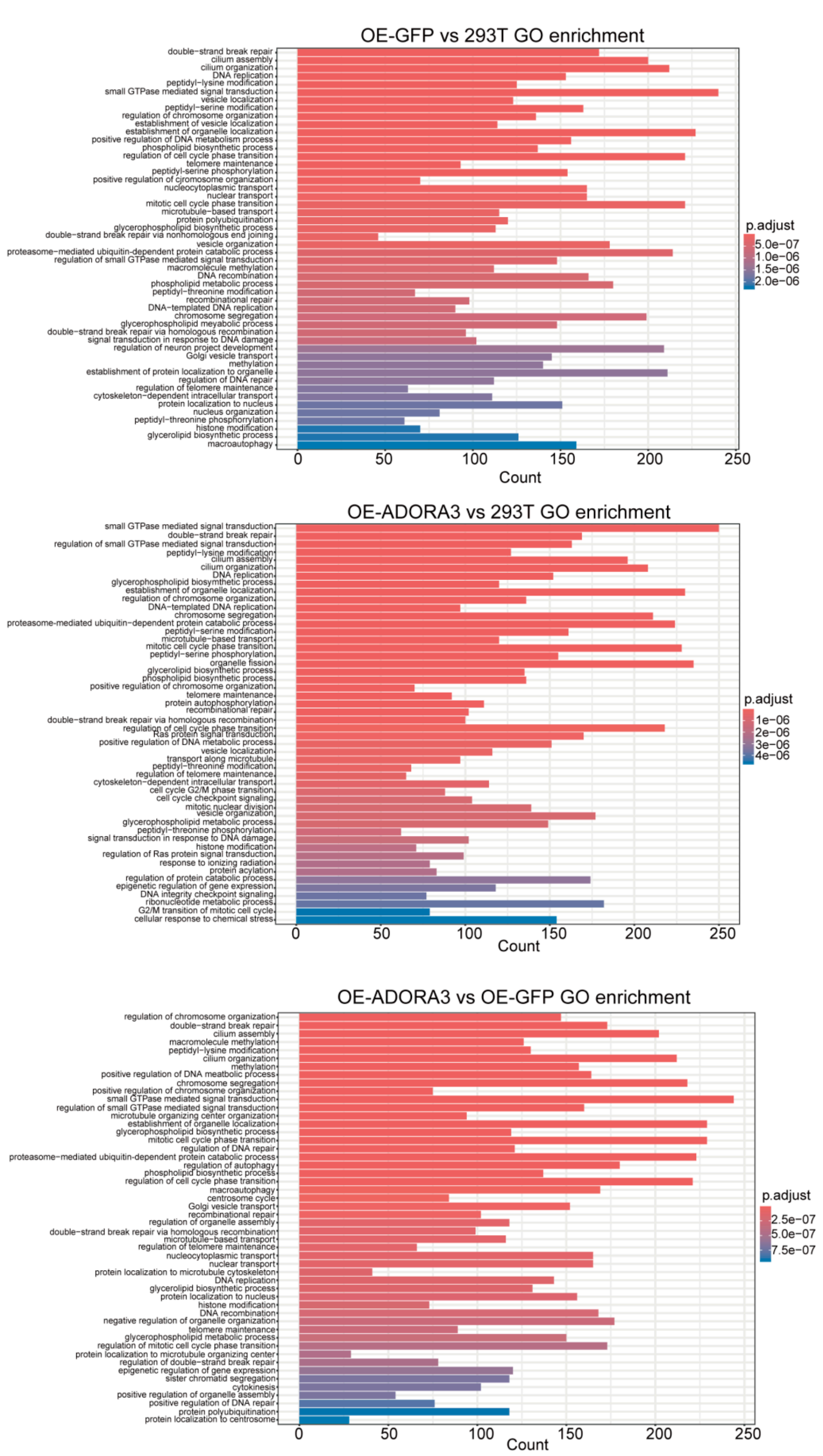

Figure. S 3 Top fifty enrichment GO terms of gDTUs between. (A), OE-GFP and 293T (B), OE-ADORA3 and 293T (C), OE-ADORA3 and OE-GFP.

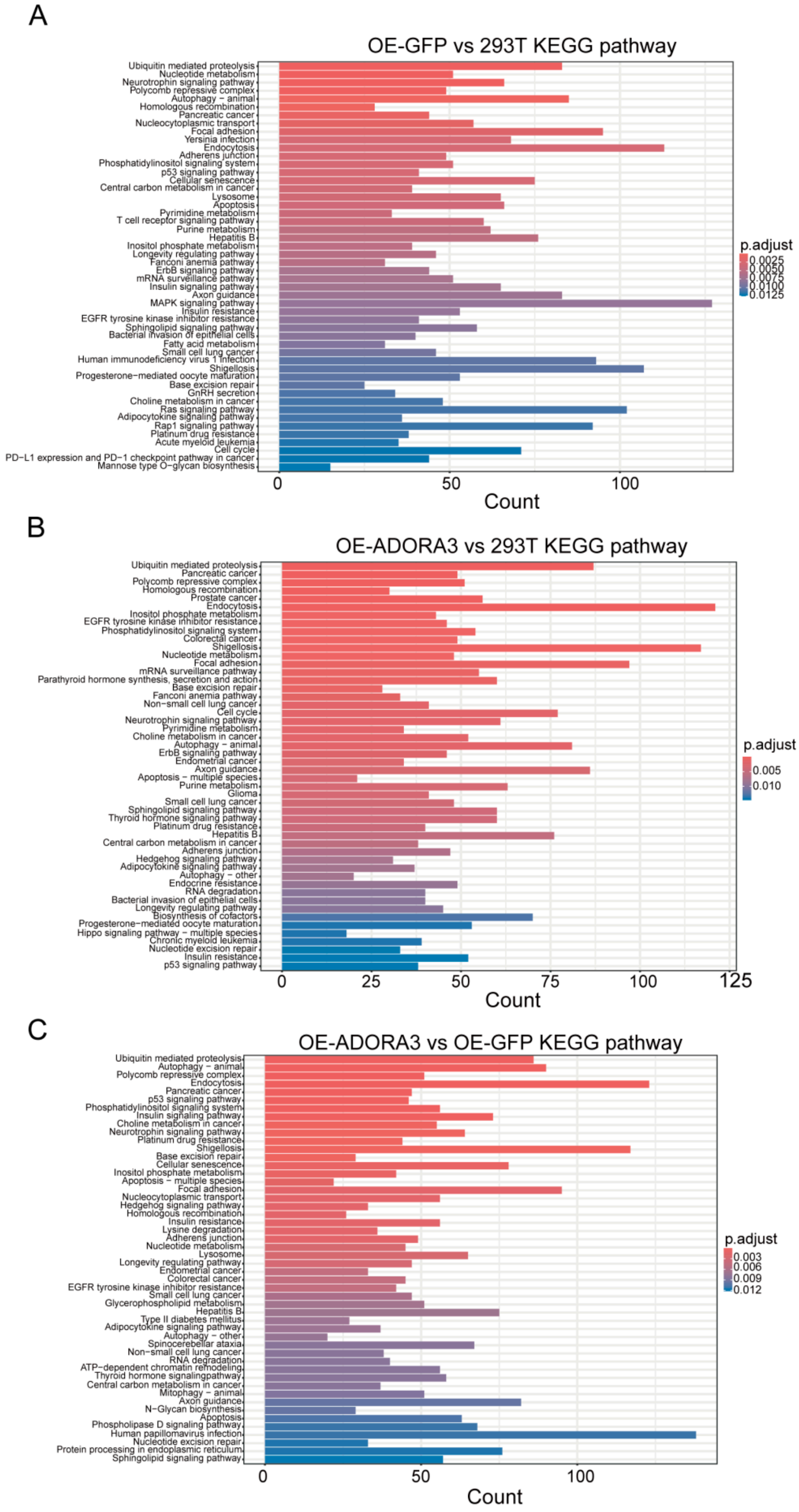

Figure. S 4 Top fifty enriched KEGG pathways of gDTUs between. (A), OE-GFP and 293T (B), OE-ADORA3 and 293T (C), OE-ADORA3 and OE-GFP.

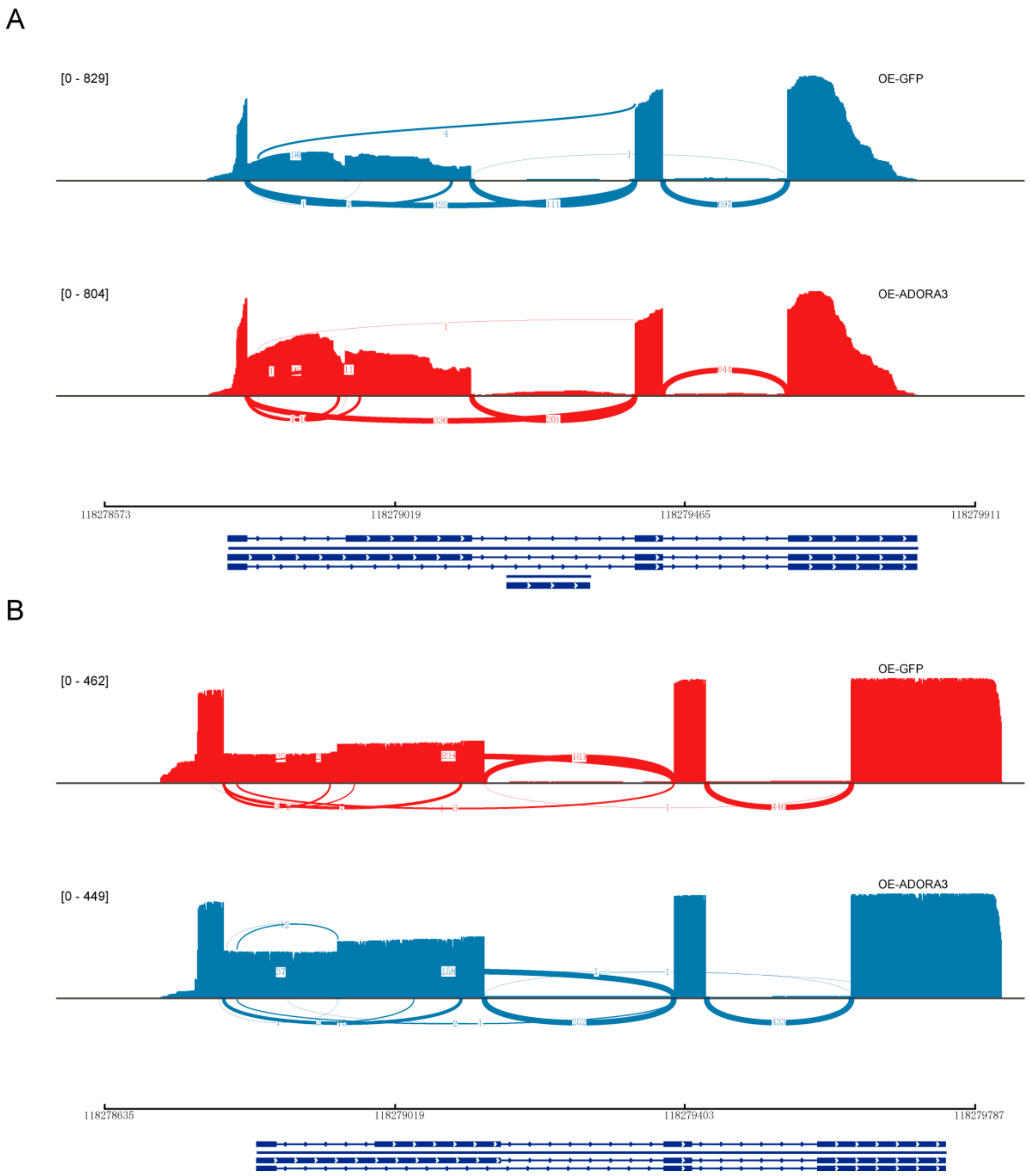

Figure. S 5 SNHG8's sashimi plot of NR_034011, NR_034010, and NR_003583 visualized in IGV in OE-GFP and OE-ADORA3. (A), short reads (B), the ONT reads.

Supplement Tables

Table. S 1 Number of transcripts per million of DEGs in OE-GFP vs 293T

Table. S 2 Number of transcripts per million of DEGs in OE-ADORA3 vs 293T

Table. S 3 Number of transcripts per million of DEGs in OE-ADORA3 vs OE-GFP

Table. S 4 gDTU in OE-GFP vs 293T

Table. S 5 gDTU in OE-ADORA3 vs 293T

Table. S 6 gDTU in OE-ADORA3 vs OE-GFP
